# Detection of crossed cerebellar diaschisis in hyperacute ischemic stroke using arterial spin-labeled MR imaging

**DOI:** 10.1371/journal.pone.0173971

**Published:** 2017-03-21

**Authors:** Koung Mi Kang, Chul-Ho Sohn, Seung Hong Choi, Keun-Hwa Jung, Roh-Eul Yoo, Tae Jin Yun, Ji-hoon Kim, Sun-Won Park

**Affiliations:** 1 Institute of Radiation Medicine, Seoul National University Medical Research Center, Seoul, Republic of Korea; 2 Department of Radiology, Seoul National University Hospital, Seoul, Republic of Korea; 3 Department of Neurology, Clinical Research Institute, Seoul National University Hospital, Seoul, Republic of Korea; 4 Department of Radiology, Seoul National University Boramae Hospital, Seoul, Republic of Korea; "INSERM", FRANCE

## Abstract

**Background and purpose:**

Arterial spin-labeling (ASL) was recently introduced as a noninvasive method to evaluate cerebral hemodynamics. The purposes of this study were to assess the ability of ASL imaging to detect crossed cerebellar diaschisis (CCD) in patients with their first unilateral supratentorial hyperacute stroke and to identify imaging or clinical factors significantly associated with CCD.

**Materials and methods:**

We reviewed 204 consecutive patients who underwent MRI less than 8 hours after the onset of stroke symptoms. The inclusion criteria were supratentorial abnormality in diffusion-weighted images in the absence of a cerebellar or brain stem lesion, bilateral supratentorial infarction, subacute or chronic infarction, and MR angiography showing vertebrobasilar system disease. For qualitative analysis, asymmetric cerebellar hypoperfusion in ASL images was categorized into 3 grades. Quantitative analysis was performed to calculate the asymmetric index (AI). The patients’ demographic and clinical features and outcomes were recorded. Univariate and multivariate analyses were also performed.

**Results:**

A total of 32 patients met the inclusion criteria, and 24 (75%) presented CCD. Univariate analyses revealed more frequent arterial occlusions, higher diffusion-weighted imaging (DWI) lesion volumes and higher initial NIHSS and mRS scores in the CCD-positive group compared with the CCD-negative group (all p < .05). The presence of arterial occlusion and the initial mRS scores were related with the AI (all p < .05). Multivariate analyses revealed that arterial occlusion and the initial mRS scores were significantly associated with CCD and AI.

**Conclusion:**

ASL imaging could detect CCD in 75% of patients with hyperacute infarction. We found that CCD was more prevalent in patients with arterial occlusion, larger ischemic brain volumes, and higher initial NIHSS and mRS scores. In particular, vessel occlusion and initial mRS score appeared to be significantly related with CCD pathophysiology in the hyperacute stage.

## Introduction

Diaschisis refers to secondary neuronal depression in an area of the brain caused by loss of connections with a remote injured brain area [1]. Crossed cerebellar diaschisis (CCD) is defined as decreased blood flow and metabolism contralateral to a damaged supratentorial area [2]. The most common mechanism of CCD has been suggested to involve disruption of the corticopontocerebellar tract [2–4]. Previous studies have suggested that CCD occurs secondary to supratentorial infarction and that it is a prognostic indicator of neurological improvement and clinical outcomes after infarction [5–8]. Therefore, it is necessary to identify a simple, noninvasive method to detect and intensively study CCD.

Since Baron et al first described CCD in a PET study [9], most studies have used positron emission tomography (PET) or single photon emission computed tomography (SPECT) to detect CCD [2,6,8,10–14]. Some studies have examined CCD using dynamic susceptibility contrast (DSC) perfusion MRI [15–17], but this method requires an intravenous injection of an exogenous MR contrast media. Arterial spin-labeling (ASL) is becoming increasingly used as a completely noninvasive perfusion-weighted MRI technique to evaluate cerebral hemodynamics. Because ASL uses endogenous arterial water as a freely diffusible tracer (instead of exogenous radioisotopes), it represents a noninvasive alternative to SPECT and PET for studying CCD [18,19].

Recently, a prospective study using ASL reported a 52% CCD detection rate of the subacute stage in ischemic stroke, which is in line with the results of a PET/SPECT series [20]. In addition, we previously reported that the asymmetric index (AI) of CCD obtained using ASL was significantly correlated with the AI obtained using SPECT, suggesting that ASL could be used as a noninvasive alternative to SPECT for evaluating CCD [21]. Therefore, in the previous study, ASL was validated both against a gold-standard perfusion method (i.e., SPECT) and for its ability to detect CCD.

Thus far, most studies have assessed CCD in subacute to chronic infarctions. Although some studies using SPECT and PET have noted that CCD can occur in hyperacute middle cerebral artery (MCA) territory infarctions [8,11], the exact frequency of CCD in hyperacute ischemic stroke is unknown. In addition, while the development of CCD in acute stroke has been shown to be closely related to the volume of supratentorial hypoperfusion or the location of infarction [4,8,10,11], the pathophysiology and relevant clinical factors of CCD in hyperacute stroke have never been studied. The purposes of this study were to evaluate the ability of ASL perfusion imaging to detect CCD in patients with first unilateral supratentorial hyperacute stroke and to identify the relevant imaging or clinical factors of CCD development.

## Materials and methods

This study was approved by the institutional review board of the Seoul National University Hospital. The institutional review board waived the need for written informed consent from the participants due to the retrospective nature of this study.

### Subjects

In a review of our radiology database between October 2014 and July 2015, we identified 204 consecutive patients who visited our hospital less than 8 hours after the onset of stroke symptoms and who underwent hyperacute stroke MRI upon admission. Patients were excluded for the following reasons: (1) no diffusion restriction region (n = 95) [22,23]; (2) any abnormality in the cerebellum or brain stem on fluid-attenuated inversion recovery (FLAIR) or diffusion-weighted imaging (DWI) (n = 30); (3) bilateral supratentorial diffusion-restricted lesions (n = 9); (4) subacute or chronic infarction (n = 22); (5) vertebrobasilar disease on MR angiography (n = 2); (6) poor-quality ASL images (n = 10); or (7) MRI using machines other than a Discovery MR750w 3.0T (n = 4).

Patient demographic data, stroke risk factors, last known normal time, stroke pathogenesis and the use of tissue plasminogen activator (tPA) before MRI examination were systematically recorded for all patients after completion of the diagnostic work-ups [24]. Stroke severity was assessed at the time of the admission, at discharge, and after 3 months using the National Institutes of Health Stroke Scale (NIHSS), with scores ranging from 0 (normal) to 42 (death), and the modified Rankin Scale (mRS), with scores ranging from 0 (normal) to 6 (death). The mRS score before the stroke episode was also determined for all patients.

### MRI protocol

All patients underwent MR examination using a 3.0-T unit (Discovery MR750w 3.0T; GE Medical Systems, Milwaukee, WI, USA) with a 32-channel head coil. The imaging protocol for hyperacute stroke included DWI (b value = 0, 1000 sec/mm^2^), FLAIR, DSC perfusion, ASL perfusion, and 3-dimensional time-of-flight MR angiography. The MRI protocol for hyperacute stroke with no contrast included the same sequences except for DSC perfusion.

For DWI and perfusion lesion volumes, DWI and DSC perfusion were processed using commercially available software approved by the Food and Drug Administration (Olea Sphere; Olea Medical SAS, La Ciotat, France), and T_max_ maps were automatically generated. A block-circulant singular value decomposition technique was used to perform the DSC analysis [25]. For the DWI lesion volume, a map of the infarction was generated using a threshold method (apparent diffusion coefficient < 600×10^−6^ mm^2^/s) [26]. For perfusion lesion volume, regions of hypoperfusion were defined as T_max_ > 6 seconds [27].

ASL perfusion imaging was performed using a 3D pseudo continuous ASL pulse sequence provided by GE Healthcare. ASL images were acquired for 2 seconds of labeling followed by 1.525 seconds of labeling delay. Background suppression was performed by using saturation pulses with crusher gradients applied below the labeling plane, allowing for an increase in the sharpness of the bolus [28]. The image acquisition consisted of a stack of interleaved 3D fast spin echo spiral readouts. Each spiral arm included 512 sampling points in the k-space, and a total of 8 interleaves (arms) were separately acquired. In addition, reconstruction was performed with the following parameters: section thickness, 5 mm; intersection gap, 0 mm; sections, 30; field of view, 240 x 240 mm; and matrix, 128 x 128, by using a Fourier transform algorithm after the k-space data were regridded (TR, 4446 ms; TE, 9.9 ms; number of excitations, 2). The signal intensity change between the labeled image and the control image was fitted to a model, from which a quantitative perfusion map of CBF was obtained. Fermi windowing for ringing artifact reduction was used to filter the images, and grad warp was not applied. The scan time was 3 minutes and 11 seconds. The detailed acquisition parameters for any sequence other than ASL are described in S1 Table.

### Image analysis

We used two methods for evaluating CCD: a qualitative analysis for the presence or absence of CCD (CCD positive and CCD negative groups) and a quantitative analysis for the degree of asymmetry of cerebellar perfusion using the AI.

#### Qualitative analysis

**Fig 1:**
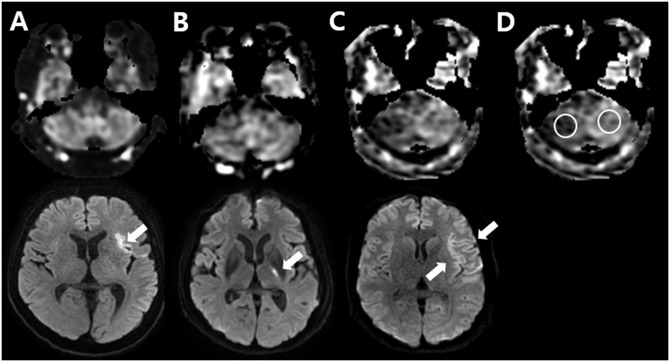
Representative ASL images for each visual grade (upper row) and diffusion-weighted images showing supratentorial infarction for each case (lower row). (A) Grade I, no demonstrable asymmetric perfusion in the cerebellum. Diffusion-weighted image in the lower row shows hyperacute infarction in the left temporal lobe (arrow). (B) Grade II, the affected right cerebellum is slightly hypointense to the unaffected left cerebellum. Hyperacute infarction is seen in the posterior limb of the left internal capsule (arrow). (C) Grade III, the affected right cerebellum is markedly hypointense to the unaffected left cerebellum. Diffusion-weighted image in the lower row demonstrates hyperacute infarction in the left frontotemporal lobe (arrow). (D) An example of circular region of interests in the cerebellum. The calculated asymmetry index was 3.7 in *A*, 9.5 in *B*, and 34.2 in *C*.

To detect CCD, a qualitative analysis was performed using the CBF map from ASL imaging. Two radiologists with 7 and 27 years of experience in neuroradiology who were blinded to CCD status and stroke location evaluated the cerebellum from bottom to top [21]. The signal intensity of the affected cerebellum was assigned one of the following 3 grades: grade I, in which the affected cerebellum was isointense to the unaffected cerebellum; grade II, in which the affected cerebellum was slightly hypointense to the unaffected cerebellum; and grade III, in which the affected cerebellum was markedly hypointense to the unaffected cerebellum (Fig 1). A grade of II or III was considered a positive CCD diagnosis [21]. For the CCD-positive group, the laterality of the cerebellar hypoperfusion was checked whether it was contralateral to the supratentorial stroke or not.

#### Quantitative analysis

For the quantitative analysis, the CBF map based on ASL was used to assess the AI. Circular regions of interest measuring 25 mm in diameter were manually drawn on the affected and mirrored cerebellar hemispheres (Fig 1). The degree of CCD was measured on a slice of an axial scan representing the greatest cerebellar asymmetry [20]. The AI between the affected cerebellar hemisphere (A) and unaffected cerebellar hemisphere (U) was calculated as follows [8,15,16]:

AI={(U−A)÷U}×100%

To assess the reproducibility of the measurements, the AI was measured twice by the same reader at 2-week intervals. The mean value of the repeated measurements was used to determine the AI.

All images were analyzed with respect to the following: (1) presence or absence of arterial occlusion on MR angiography; (2) DWI lesion volume; (3) perfusion lesion volume; and (4) perfusion-diffusion mismatch ratio.

### Statistical analysis

To assess inter-observer agreement for the presence of CCD in the qualitative analysis, the kappa statistic was used [21]. κ values of < 0.20, 0.21–0.40, 0.41–0.60, 0.61–0.80, and > 0.81 indicated poor, fair, moderate, good and excellent agreement, respectively. Intra-observer reproducibility for the AIs was assessed by calculating intraclass correlation coefficients (ICCs) [29]. ICCs of < 0.40, 0.40–0.59, 0.60–0.74, and > 0.75 indicated poor, fair, good and excellent reproducibility, respectively, [30]. We also performed a receiver operating characteristic (ROC) analysis to evaluate the diagnostic performance of the AI compared with that of visual grading of CCD.

To compare the CCD-positive and CCD-negative groups, descriptive data were analyzed using Fisher’s exact test for categorical variables, and the Mann-Whitney U test was used to analyze non-categorical data. Thereafter, multivariate stepwise logistic regression analysis was performed to identify factors independently associated with CCD using p < .1 in the univariate analysis, which was considered to indicate potential factors associated with CCD [31].

Regression analysis was used for AI and all clinical and MR imaging factors. All quantitative variables were included in the linear regression as continuous variables. All variables with P values of < .1 based on simple regression were then evaluated by stepwise multiple regression analyses to identify factors independently associated with AI.

Statistical analyses were performed with commercially available software (SPSS, version 20.0 for Windows, SPSS, Chicago, Ill; and MedCalc, version 9.3.0.0, MedCalc Software, Mariakerke, Belgium). p < .05 was considered to indicate a statistically significant difference.

## Results

Thirty-two patients with first hyperacute unilateral supratentorial ischemic stroke were enrolled in this study. All patients underwent MRI within a median time of 140 minutes (interquartile range, 110–182 minutes) after symptom onset. The patients’ baseline characteristics are provided in Table 1. One patient was excluded from perfusion lesion volume and mismatch ratio analyses because he had previously undergone non-contrast hyperacute MRI due to poor kidney function. The mRS scores of all patients before their stroke episodes were zero. A stroke severity score at 3 months was not available for 3 patients because they were transferred to other hospitals.

**Table 1:** Clinical characteristics and MRI findings between CCD-positive and CCD-negative groups.

Characteristics	All, n = 32	CCD-positive group, n = 24	CCD-negative group, n = 8	p value
Age—year †	62.6±14.0	64.6±9.4	61.9±15.2	.913
Male sex—no. (%)‡	21 (66)	15 (63)	6 (75)	.687
Median time between onset and MRI, minutes (IQR) †	139.5 (110–181.5)	139.5 (113–210.5)	137.5 (80.5–166)	.296
Risk factors—no. (%)‡				
Atrial fibrillation	12 (38)	8 (33)	4 (50)	.433
Hypertension	13 (41)	9 (38)	4 (50)	.683
Hyperlipidemia	8 (25)	4 (17)	4 (50)	.152
Diabetes mellitus	3 (9)	2 (8)	1 (13)	1
Smoking	4 (13)	2 (8)	2 (25)	.268
Cause of stroke—no. (%)‡				
Large-artery occlusion	7 (22)	6 (25)	1 (13)	1
Cardioembolic occlusion	15 (47)	10 (42)	5 (63)	.423
Small vessel occlusion	3 (9)	2 (8)	1 (13)	1
Undetermined or other	8 (25)	7 (29)	1 (13)	.642
Use of tPA—no. (%)‡	10 (31)	7 (29)	3 (28)	.681
Presence of arterial occlusion—no. (%)‡	18 (56)	17 (71)	1 (13)	.010*
Location of arterial occlusion—no. (%)‡				
ICA	7 (22)	7 (29)	0	.550
MCA	10 (31)	9 (38)	1 (13)	.380
PCA	2 (6)	2 (8)	0	1
Median DWI lesion volume, mL (IQR) †	3.36 (0.69–12.0)	5.71 (1.37–18.5)	0.63 (0.29–1.92)	.015*
Median perfusion lesion volume, mL (IQR) †	n = 31	n = 23	n = 8	
	9.48 (0.18–67.72)	28.77 (0.82–78.1)	0.99 (0.35–8.26)	.057
Perfusion-diffusion mismatch ratio†	n = 31	n = 23	n = 8	
	1.3 (0.18–5.46)	1.3 (0.18–6.51)	1.5 (0.39–4.93)	.785
Score at initial presentation				
Median NIHSS score (IQR) †	4.5 (2–13)	7 (2–13.5)	1.5 (1–4)	.049*
Median mRS score (IQR) †	3.5 (2–4)	4 (3.4)	2 (2–2.5)	.006*
Score at discharge	n = 32	n = 24	n = 8	
Median NIHSS score (IQR) †	1 (0–3)	1 (0–6)	0 (0–1)	.135
Median mRS score (IQR) †	1.5 (0–3)	2 (0–3)	0 (0–1.5)	.078
Score after 3 months	n = 29	n = 21	n = 8	
Median NIHSS score (IQR) †	0 (0–0.25)	0 (0–1.25)	0 (0–0)	.322
Median mRS score (IQR) †	0 (0–0.25)	0 (0–0)	0 (0.2)	.301

MRI, magnetic resonance imaging; CCD, crossed cerebellar diaschisis; IQR, interquartile range; tPA, tissue plasminogen activator; DWI, diffusion-weighted imaging; ICA, internal cerebral artery; MCA, middle cerebral artery; PCA, posterior cerebral artery; NIHSS, National Institutes of Health Stroke Scale; and mRS, modified Rankin Scale

^†^ Mann-Whitney U test

^‡^ Fisher’s exact test

*Significant variables for each model.

In the qualitative analysis, asymmetric cerebellar hypointensity in ASL (grade II or III) was observed in 24 of 32 patients (75%) by both observers 1 and 2. Two cases classified as grades I and II by observer 1 were classified as grades II and I by observer 2, respectively. The inter-observer agreement was excellent (κ value: 0.864). In the CCD-positive group, the cerebellar hypoperfusion was contralateral to the supratentorial stroke.

**Fig 2:**
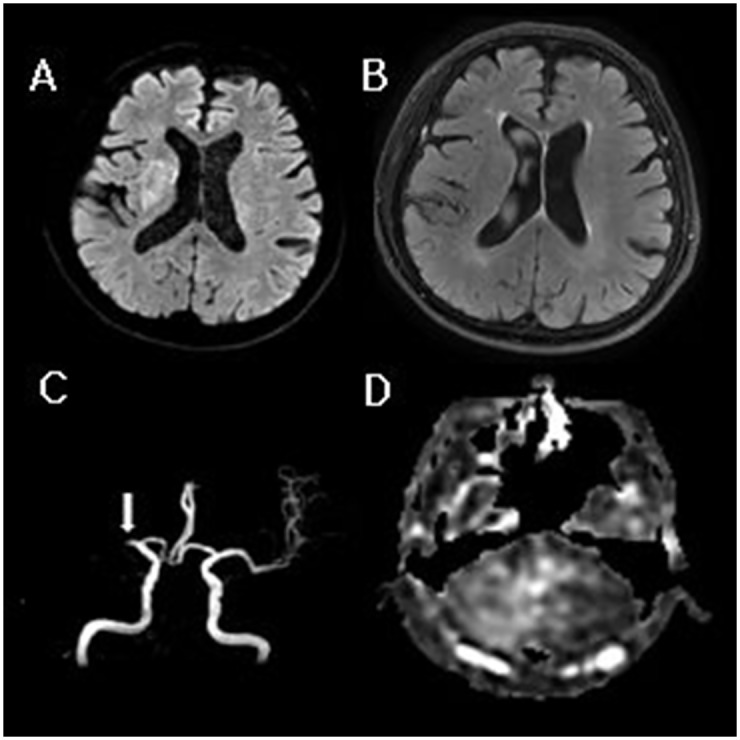
A 71-year-old man with a history of sudden onset left-sided weakness. (A) Diffusion-weighted image demonstrating hyperacute infarction in the right basal ganglia without (B) signal change on the fluid-attenuated inversion recovery image. (C) Arterial occlusion is noted in MR angiography at the right M1 (arrow). (D) ASL image of the cerebellum showing hypoperfusion in the contralateral cerebellar hemisphere (grade III and AI of 44.48).

CCD was more frequently observed in patients with arterial occlusion than in those with no occlusion (p = .01). The DWI lesion volume and initial NIHSS and MRS scores were significantly greater in the CCD-positive group than in the CCD-negative group (p = .015, p = .049, and p = .006) (Table 1). No significant differences in age, sex, time from stroke onset to MRI, risk factors, cause of stroke, or location of arterial occlusion were observed between the CCD-positive and CCD-negative groups (Table 1). The perfusion lesion volume and mismatch ratio were not significantly different between the two groups. Multivariate stepwise logistic regression analysis was then performed using the presence of arterial occlusion, DWI lesion volume, perfusion lesion volume, and initial NIHSS and MRS scores (p < .01). The results revealed that arterial occlusion and initial mRS scores were significantly associated with CCD (odds ratio 21.94, 95% confidence interval 1.42–339.52, p = .027 for arterial occlusion; odds ratio 4.14; 95% confidence interval 1.20–14.30; p = .025 for initial mRS score). Representative images of CCD in patients with hyperacute ischemic stroke are shown in Fig 2.

In the quantitative analysis, intra-observer reproducibility was excellent for AI (ICC = 0.962). In the ROC analysis, the AI demonstrated good general agreement with the results of the qualitative analysis. When the grading scale was used as the reference standard, an AI of 51% corresponded to 92% sensitivity and 100% specificity (p < .001).

Linear regression analyses revealed that the presence of arterial occlusion and the initial mRS score were significantly related with AI (p = .004 and p = .023) (Table 2). Next, diabetes mellitus (as a risk factor), large-artery atherosclerosis (as a cause of stroke), presence of occlusion, MCA occlusion (as a site of occlusion), perfusion lesion volume, perfusion-diffusion mismatch ratio, and initial mRS score were included in multivariate stepwise regression analysis (p < .01). The results revealed that arterial occlusion was the variable most significantly associated with AI in patients with hyperacute ischemic stroke (estimates, 13.11; 95% CI, 4.74 to 21.48; p = .003).

**Table 2:** Univariate linear regression analyses for asymmetric index.

Variable	Estimates	95% confidence intervals	p value
Age	.006	(-.341, .329)	.972
Sex	-1.55	(-11.42, 9.32)	.751
Time between onset and MRI	0.03	(-0.02, 0.09)	.246
Risk factors			
Atrial fibrillation	-1.98	(-11.65, 7.69)	.679
Hypertension	-3.35	(-12.83, 6.13)	.476
Hyperlipidemia	-5.97	(-16.58, 4.65)	.260
Diabetes mellitus	-14.98	(-30.01, 1.37)	.052
Smoking	-10.32	(-24.22, 3.7)	.139
Cause of stroke			
Large artery atherosclerosis	10.44	(-0.95, 21.82)	.071
Cardioembolic occlusion	-2.56	(-11.92, 6.81)	.581
Small vessel occlusion	-5.89	(-21.85, 10.47)	.457
Undetermined or other	-2.42	(-13.22, 8.39)	.651
Use of tPA	1.66	(-8.45, 11.77)	.740
Presence of arterial occlusion	12.49	(4.24, 20.73)	.004*
Location of arterial occlusion			
ICA	3.77	(-7.50, 15.05)	.499
MCA	8.18	(-1.48, 17.84)	.094
PCA	6.89	(-12.34, 26.12)	.470
DWI lesion volume	-0.004	(-0.17, -0.16)	.960
Perfusion-lesion volume	0.08	(-0.01, 0.17)	.066
Perfusion diffusion mismatch ratio	0.02	(0, 0.04)	.086
Score at initial presentation			
NIHSS score	0.4	(-0.3, 1.1)	.255
mRS score	4.88	(0.72, 9.04)	.023*
Score at discharge			
NIHSS score	.05	(-0.41, 0.52)	.819
mRS score	.24	(-2.45, 2.92)	.859
Score after 3 months			
NIHSS score	.02	(-0.47, 0.51)	.941
mRS score	.165	(-2.84, 3.17)	.911

MRI, magnetic resonance imaging; tPA, tissue plasminogen activator; DWI, diffusion-weighted imaging; ICA, internal cerebral artery; MCA, middle cerebral artery; PCA, posterior cerebral artery; NIHSS, National Institutes of Health Stroke Scale; and mRS, modified Rankin Scale

*Significant variables (p < .05)

## Discussion

A significant finding of this study was that CCD was identified in ASL images in 75% of patients who had experienced their first unilateral supratentorial hyperacute infarction. CCD was detected with excellent inter-rater agreement and quantified using AIs with excellent reproducibility. Univariate analysis of the associations of the clinical characteristics and MRI findings with the visually assessed CCD revealed that DWI lesion volume, initial NIHSS and mRS scores and arterial occlusion were correlated with the development of CCD. Furthermore, perfusion lesion volume, perfusion-diffusion mismatch ratio, initial mRS score and arterial occlusion were correlated with the measured AI. Finally, multivariate analyses revealed that arterial occlusion and initial mRS score were independent factors related to the visually assessed CCD in hyperacute stroke. In addition, arterial occlusion corresponded to an independent significant association with the measured AI.

A major strength of this study was the use of an ASL method that had been previously validated against SPECT for CCD detection [21]. This feature strengthens the impact of our findings, as any data acquired using a non-validated ASL method would not represent valuable results for CCD due to unknown performance in the posterior circulation. We observed CCD in 24 of 32 patients (75%) using ASL, and the shortest time between symptom onset and CCD was 63 minutes. Although previous studies have reported that CCD can occur in hyperacute infarctions within the first 3 hours after the onset of stroke symptoms [8,11], there have been no reports regarding the frequency of CCD in hyperacute stroke. Prior studies using SPECT, DSC-perfusion and CT-perfusion reported that the incidence of CCD in acute stroke was less than 50% [13,16,32].

In our study, arterial occlusion was the variable most strongly associated with CCD and AI. Of the 24 CCD-positive patients, 17 (71%) showed definite arterial occlusion on MR angiography, and 7 did not. Although arterial occlusion was not observed on MR angiography in these 7 patients, distal occlusion was presumed in one case because we observed vascular hyperintensity on FLAIR and susceptibility vessel signs at the M2 segment of the MCA near the infarcted areas. Additionally, one case showed severe stenosis at the left proximal ICA. Furthermore, one case exhibited possible spontaneous partial recanalization before the initial MR imaging due to the presence of residual stenosis at the right anterior cerebral artery and a hyperemic response within the area on ASL images corresponding to the diffusion-restricted area [33]. The other three cases had small infarctions in the posterior limb of the internal capsule and the corona radiata (DWI lesion volumes of 1.1 mL, 0.4 mL, and 0.5 mL).

Regarding the relationship between CCD and DWI lesion volume, previous studies using SPECT or DSC perfusion in acute infarction reported that the initial DWI lesion volume was significantly associated with the AI and that the mean volume of the DWI abnormality was significantly higher in CCD-positive cases [13,16]. Regarding the relationship between CCD and abnormal perfusion, a previous study using DSC perfusion in patients with acute infarction showed that the mean volume of the supratentorial time-to-peak abnormality was significantly higher in CCD-positive cases [16]. Additionally, a study using dynamic CT perfusion imaging in patients with acute infarction revealed that the supratentorial ischemic volume, the degree of perfusion reduction, and the AI were strongly and significantly correlated [32]. The results of our study agree with the aforementioned studies, as our univariate analysis revealed that the DWI lesion volume was significantly higher in the visually CCD-positive cases compared with the CCD-negative cases. However, perfusion lesion volume revealed a p value of 0.066 related with AI. In addition, multivariate analyses revealed that the DWI lesion volume was not independently associated with either CCD or AI. Given the small sample size of our study, the results regarding DWI lesion volume and perfusion lesion volume are exploratory, and a larger sample size should be investigated in future studies.

In our study, multivariate analyses revealed that the initial mRS score was independently related to the presence of CCD and that it had a marginally significant association with the AI. However, there was no significant difference in the stroke severity scores at the time of follow-up between the CCD-positive and CCD-negative groups. Additionally, no significant relationship was observed between the stroke severity scores at the 3-month follow-up and the AI. These findings are in agreement with the results of previous studies using SPECT or PET, which demonstrated contralateral cerebellar hypoperfusion on early scans but that CCD at the earlier time points was not correlated with the clinical scores at later time points [5,7,8,34]. Therefore, according to these investigations, the presence and degree of CCD in hyperacute stroke do not appear to be suitable predictors of disease outcome. However, it must be noted that most of the cohort showed neurologic improvement at follow-up. The median NIHSS and mRS scores at discharge were 1 and 2 in the CCD-positive group, and both scores were 0 in the CCD-negative group, whereas at the 3-month follow-up, the median NIHSS and mRS scores for both groups were all 0. In light of this evidence, further studies including patients with various prognoses are warranted to evaluate the potential of CCD as a biomarker of hyperacute stroke.

The present study has several limitations. First, this was a retrospective study, and the possibility of selection bias cannot be excluded. However, because our hospital required patients clinically suspected of having an ischemic episode to undergo immediate hyperacute stroke MRI within a short timeframe, we assumed that the vast majority of patients with hyperacute ischemic infarction were included in this study. Second, the sample size was small, and the distribution was skewed. Therefore, it is difficult to make a final conclusion based on the results presented here. Although our study provides valuable data on consecutively admitted patients, a further study including a larger cohort is required to generalize our results. Third, we used MR angiography as the reference standard to confirm arterial occlusion because conventional digital subtraction angiography was performed on a limited number of patients who met the indications for intra-arterial thrombolysis. Thus, there was a CCD-positive case in which peripheral occlusion was not delineated on MR angiography, although distal occlusion was suspected on FLAIR and susceptibility-weighted images. Fourth, although all of the patients underwent MRI as soon as possible after admission, spontaneous recanalization was possible before the initial MRI, which might have resulted in underestimation of the prevalence of arterial occlusion. Fifth, because ASL is technique dependent, it is important to note that the AI can vary according to the protocol and equipment used when considering the use of this technique for follow-up. The ordinal validation of the sequence was performed on 1.5T GE scanners in the previous report [21]. In the present work, a 3T GE scanner was used for the same sequence. Different parameters are typically used on 3T scanners; however, because higher field strength scanners carry additional benefits, including improved the signal intensity–to-noise ratio and greater background suppression, similar or better performance using 3T scanners can be expected.

In conclusion, in the present study, we detected a high frequency (75%) of CCD in patients with hyperacute ischemic stroke using ASL imaging. We found that CCD was more prevalent in patients with arterial occlusion, larger ischemic brain volumes, and higher initial NIHSS and mRS scores. In particular, the presence of CCD and the measured AI were significantly associated with arterial occlusion and initial mRS scores. Therefore, the presence of CCD in hyperacute ischemic stroke may imply a greater probability of arterial occlusion. In addition, CCD in patients with hyperacute ischemic stroke could be associated with initial functional dependence.

## Supporting information

S1 Table MR imaging parameters. (DOCX)

S1 File Dataset. (XLSX)
