# Supplementary material for: Detection of crossed cerebellar diaschisis in hyperacute ischemic stroke using arterial spin-labeled MR imaging
Source: PLoS One. 2017 Mar 21;12(3):e0173971. doi: 10.1371/journal.pone.0173971 (PMC5360263; doi:10.1371/journal.pone.0173971)
Supplement: S1 Table — (DOCX) [file pone.0173971.s001.docx]

**S1 Table. MR Imaging parameters**

| **Parameters** | **DWI** | **TOF MRA** | **FLAIR** | **DSC** |
| --- | --- | --- | --- | --- |
| Repetition time (msec) | 6000 | 19 | 9200 | 1450 |
| Echo time (msec) | 73 | 3.4 | 130 | 22 |
| Flip angle (degree) |  | 18 | 160 | 90 |
| Section thickness (mm) | 3.6 | 1.0  (recon 0.5) | 5.0 | 4.0 |
| Intersection gap (mm) | 0.4 | n/a | 1.0 | 0 |
| FOV (mm) | 240 | 220 | 220 | 220 |
| Matrix | 160x160 | 320x192 | 320x192 | 128x128 |
| No. of signals acquired | 2 | 1 | 1 | 1 |
| Etc | B=1000 |  | TI=2280.66 | Multiphase= 60 |
|  |  |  |  | Gadolinium contrast agent 0.1mmol/Kg rate 5mL/s |

* DWI indicates diffusion-weighted imaging; TOF MRA, time-of-flight MR angiography imaging; FLAIR, fluid-attenuated inversion recovery; DSC, dynamic susceptibility contrast perfusion imaging; FOV, field of view; No., number. and TI, inversion time
